# Comparison of sputum induction and bronchoscopy in diagnosis of sputum smear-negative pulmonary tuberculosis: a systemic review and meta-analysis

**DOI:** 10.1186/s12890-020-01192-w

**Published:** 2020-05-25

**Authors:** Wen Luo, Yihua Lin, Zhibin Li, Wanyu Wang, Yonghong Shi

**Affiliations:** 1 Department of Pulmonary and Critical Care Medicine, the First Affiliated Hospital of Xiamen University, Xiamen, 361001 China; 2 Epidemiology Research Unit, the First Affiliated Hospital of Xiamen University, Xiamen, China

**Keywords:** Tuberculosis, Induced sputum, Bronchoscopy, Meta-analysis, Diagnosis

## Abstract

**Background:**

Pulmonary tuberculosis is one of the most common infectious diseases worldwide. Patients with suspected pulmonary tuberculosis with negative smear are recommended to undergo further tests including sputum induction and bronchoscopy. Our study is aimed to compare sputum induction and bronchoscopic specimens in the diagnosis of sputum smear-negative pulmonary tuberculosis.

**Method:**

PubMed, Web of Science, Cochrane Library and Embase were searched for eligible studies. The pooled sensitivities (SEN), specificities (SPE), positive likelihood ratio (PLR), negative likelihood ratio (NLR), diagnostic odds ratio (DOR) and 95% confidence intervals (CI) were constructed, and the areas under the curves (AUCs) were calculated.

**Results:**

Five studies with a total number of 586 cases were included. For mycobacterial culture, the SEN and SPE of sputum induction were 0.72(95% CI, 0.66–0.77) and 1.00(95%CI, 0.99–1.000) respectively, whereas the SEN and SPE of bronchoscopy were 0.70(95%CI, 0.64–0.75) and 1.00(95%CI, 0.99–1.00) respectively. Sputum induction had a similar AUC (0.9564, SE = 0.0749) with bronchoscopy (0.8618, SE = 0.1652) (*P* = 0.602). For specimen of acid-fast bacilli smear, the SEN and SPE of sputum induction were 0.35(95% CI, 0.29–0.42) and 0.99(95% CI, 0.96–1.00) respectively, whereas the SEN and SPE of bronchoscopy were 0.38(95% CI, 0.32–0.45) and 0.99(95% CI, 0.96–1.00) respectively. There is no statistically significant difference in the AUC for sputum induction (0.6016) compared with bronchoscopy (0.8163) (*P* = 0.792).

**Conclusions:**

For the diagnosis of sputum smear-negative pulmonary tuberculosis, the diagnosis yield of sputum induction and bronchoscopy is similar.

## Background

Pulmonary tuberculosis (TB) is one of the most common infectious disease worldwide and also one of the top 10 causes of death, especially in developing countries [1]. Early diagnosis is the most effective pulmonary tuberculosis control strategy because the early appropriate treatment renders these patients noninfectious and interrupts the chain of disease transmission. Acid-fast bacilli in sputum is recommended as the preliminary diagnostic method by the World Health Organization (WHO). However, the sensitivity of this method is low and the value in patients who cannot produce sputum spontaneously is very little [2, 3]. It follows that the active respiratory specimens collection is an important strategy to early diagnosis of pulmonary tuberculosis [4].

Sputum induction and/or bronchoscopy are commonly used for the diagnosis in patients with suspected tuberculosis who do not produce sputum or have a negative acid-fast bacilli smear from spontaneous sputum. Sputum induction is a safe and effective method in obtaining specimens for acid-fast bacilli smear and mycobacterial culture [5]. In areas where bronchoscopy is not readily available, sputum induction offers an alternative or additional approach to the diagnosis of sputum smear-negative pulmonary tuberculosis [6]. Bronchoscopy is more invasive, more expensive, and less-tolerated than sputum induction, but bronchoscopy can provide specimens from the lesion area of the lung.

Over the past decade, several studies [5–9] have described the diagnosis yield of sputum induction in comparison with bronchoscopy in the sputum smear-negative pulmonary tuberculosis. Because of the heterogeneous populations and small sample sizes, the results of these studies were variable. This study is aimed to compare the sensitivity and specificity of sputum induction and bronchoscopy in the diagnosis of sputum smear-negative pulmonary tuberculosis by the method of meta-analysis [5–9].

## Methods

This study was performed according to the Preferred Reporting Items for Systematic Reviews and Meta-Analyses (PRISMA) statement. The protocol for this meta-analysis is available in PROSPERO (CRD42019133766).

### Search for trials

PubMed, Web of Science, Cochrane Library and Embase up to Mar 31, 2019 were searched by two investigators independently using search terms included “tuberculosis”, “sputum induction”, “induced sputum”, “bronchoscopy” and “bronchoalveolar” to identify studies that met the inclusion criteria (see Additional file 1: Appendix S1, for complete search descriptions). There were no restrictions on language.

### Selection criteria

Studies were selected based on the following inclusion criteria: (1) sputum induction and bronchoscopy were used to detect pulmonary tuberculosis in the same patient cohorts. The test result of induced-sputum specimens was the experimental group, whereas the test result of bronchoscopic specimens was regarded as the control group; (2) enough data to calculate the outcome data (true positive (TP), false positive (FP), true negative (TN), false negative (FN)); (3) the participants were diagnosed using the gold standard; (4) the gold standard for diagnosis of pulmonary tuberculosis [10] was defined in the study; (5) sputum induction and bronchoscopy had to be performed at the time of clinical presentation with suspected tuberculosis before administration of anti-tuberculosis therapy. The exclusion criteria were as follows: (1) the diagnostic method for tuberculosis did not include sputum induction and bronchoscopy; (2) reviews, case reports, letters, proceedings, or commentaries.

### Data extraction

Two researchers independently extracted the following information from each study: name of study, first author, publication year, country, source of patients, the number of specimens collected, the concentrations of hypertonic saline, the type of nebulizers, the culture techniques, study type, sample size, reference standard, total number of TB diagnosis, type of bronchoscopic specimens, and outcome data (TP, FP, FN, and TN). Discrepancies were resolved by consensus.

### Risk-of-bias assessments

Two researchers independently used the Quality Assessment of Diagnostic Accuracy Studies (QUADAS-2) tool, which was provided by RevMan (version 5.3, Cochrane Collaboration, Oxford, UK), to assess the risk of bias and applicability of diagnostic accuracy for the studies included. There were four sections in the QUADAS-2: patient selection, index test, reference standard, and flow and timing [11]. According to the following criteria, we judged the included studies as low risk, high risk or unclear bias: (1) if all the questions for a section were replied with “yes”, then the risk of bias was judged as “low”; (2) if any question in a section was replied with “no”, then risk of bias was judged as “high”; (3) when insufficient information was provided, the risk of bias was judged as “unclear bias”. Meanwhile, we graded the applicability as low, high, or unclear with the above criteria.

### Statistical analysis

We carried out a fixed-effects model to calculate pooled results and corresponding 95% confidence intervals (CI) when there was no significant heterogeneity (*P* value of Cochran-Q of DOR > 0.1); otherwise, we applied a random-effects model. Then we constructed the pooled sensitivities (SEN), specificities (SPE), positive likelihood ratio (PLR), negative likelihood ratio (NLR), diagnostic odds ratio (DOR), and 95% confidence intervals (CI) and afterwards calculated the areas under the curves (AUCs). We conducted Z tests to compare the diagnostic accuracies of sputum induction and bronchoscopic specimens directly. To calculate the degree of heterogeneity across studies, we selected the *I*^*2*^ value and Q statistic of the chi-square test (25–50%, low heterogeneity; 51–75%, medium heterogeneity; greater than 75%, high heterogeneity) [12]. We used Deeks’ funnel plot asymmetry test to assess the publication bias, and a *P* value below 0.05 suggested the present of publication bias [13]. All statistical analyses were performed using Meta-DiSc Version 1.4, Review Manager Version 5.3, Stata Version 15 and R3.5.3 [14].

## Results

### Search results and study characteristics

**Fig. 1:**
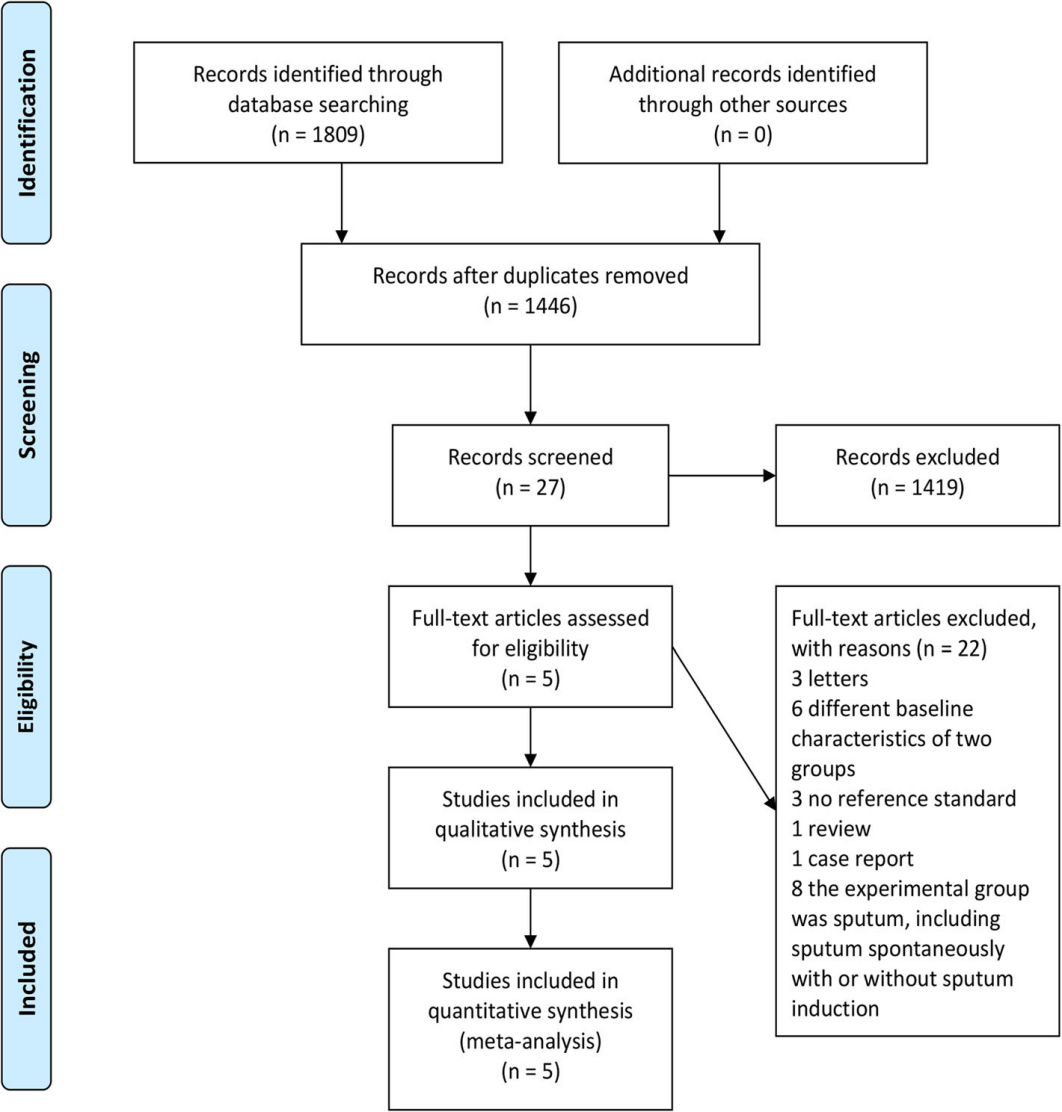
Procedure used for selection of studies (PRISMA flow diagram)

**Fig. 2:**
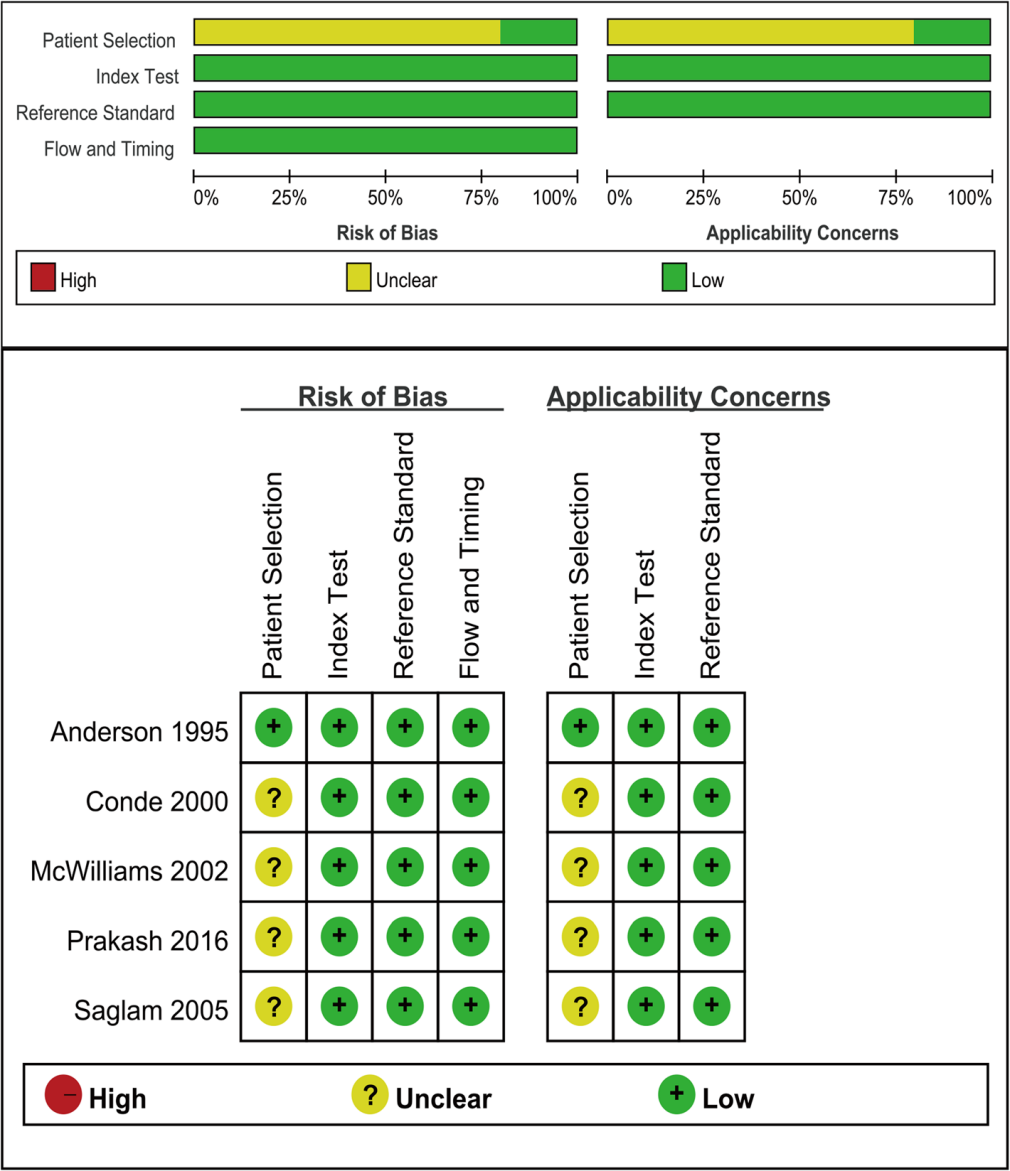
Quality assessment of the included studies (QUADAS-2)

The systematic literature searches identified 1809 potentially relevant studies. One thousand seven hundred and eighty two of these studies were eliminated before the full text assessment. The reasons of exclusion included duplicates, unrelated with the topic of the research, conference abstracts, reviews, case reports and letters. Twenty-seven records were screened in full-text articles and five qualified studies were included at last. The selection process was shown in Fig. 1. The five qualified studies [5–9] included a total of 586 cases. All the studies had sufficient data to quantitative synthesis for the mycobacterial culture of induced-sputum specimens and bronchoscopic specimens in the diagnosis of pulmonary tuberculosis, but only three studies had sufficient data on acid-fast bacilli smear [5–7]. Characteristics of those studies were presented in Table 1. The quality of all the included studies, in terms of risk of bias and applicability concerns, was acceptable according to QUADAS-2 results (Fig. 2).

**Table 1:** Characteristics of the 5 studies included in the meta-analysis

author	year	Country	Source of patients	the number of specimens collected	the concentrations of hypertonic saline	the type of nebulizers	the culture techniques	Study type	Sample size	reference standard	Total TB diagnosis	type of bronchoscope specimens
Anderson [7]^a^	1995	Canada	tertiary health care unit	once	3%	DeVilbiss ultrasonic nebulizer	Middlebrook agar or Bactec Culture Systems	PS	101	1 or 2	27	BB1,BB2,BAL。
Conde [6]^a^	2000	Brazil	primary health units or tertiary health care unit	once	3%	DeVilbiss ultrasonic nebulizer	Löwenstein–Jensen and Sabouraud’s medium	PS	251	1 or 2	143	BAL
McWilliams [8]	2002	New Zealand	Tuberculosis specialist hospital	three	3%	DeVilbiss ultrasonic nebulizer	Bactec 12B and Lowenstein-Jensen media.	PS	129	1	27	BAL, BW
Saglam [5]^a^	2005	Turkey	tertiary health care unit	once	3%	ultrasonic nebulizer	Lowenstein-Jensen medium	PSM	55	1 or 2	49	BAL
Prakash [9]	2016	India	tertiary health care unit	once	3%	nebuliser	LowensteinJensen (L-J) medium	unclear	50	1	35	BAL

^a^: Sufficient data for both Mycobacterial culture and acid-fast bacilli smear

*PS* prospective study, *PMS* Prospective multicenter study

1 = positive culture for *Mycobacterium tuberculosis*. 2 = unequivocal radiographic improvement after adequate treatment with anti-tuberculosis drugs

*BAL* bronchoalveolar lavage, *BW* bronchial washing, *BB1* bronchial brushings, *BB2* Bronchial biopsies

### Quantitative synthesis of mycobacterial culture

**Fig. 3:**
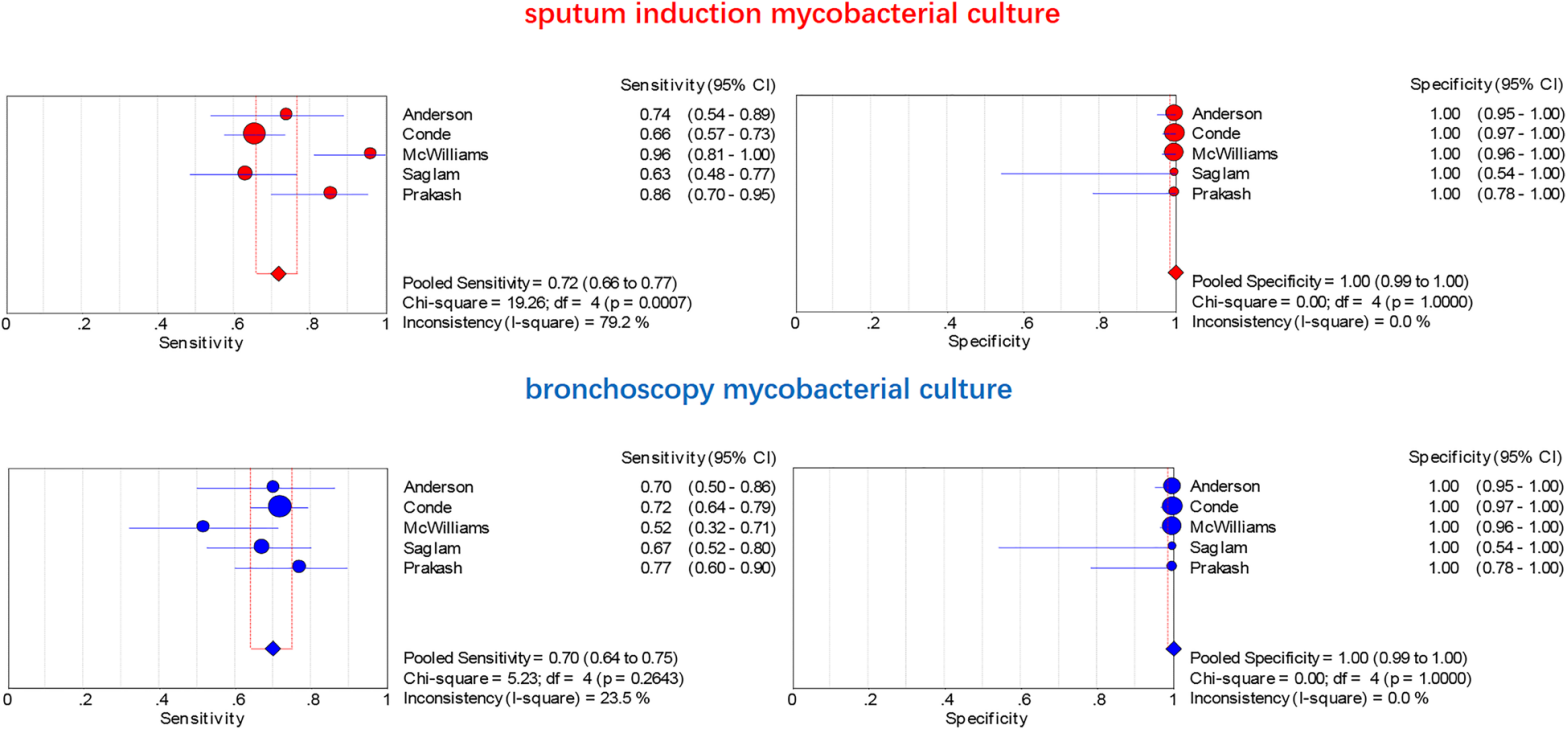
SEN and SPE of sputum induction VS bronchoscopy on mycobacterial culture

**Fig. 4:**
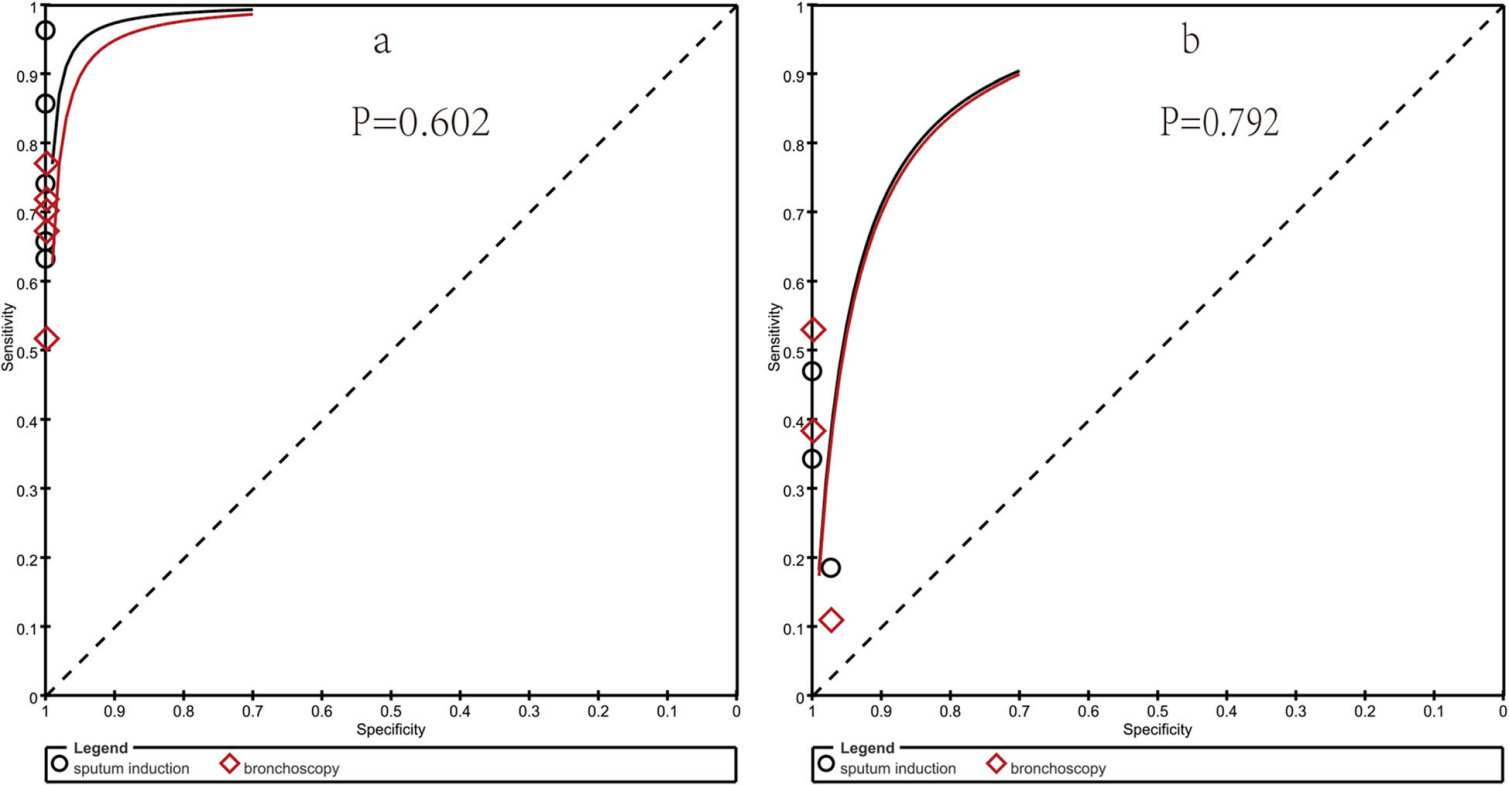
ROC of sputum induction VS bronchoscopy on mycobacterial culture(**a**) and acid-fast bacilli smear(**b**)

The Spearman correlation coefficient and *P* value of sputum induction were − 0.300 and 0.624 respectively, while the Spearman correlation coefficient and *P* value of bronchoscopy were 0.000 and 1.000, both of which indicated that there was no significant threshold effect. There was little heterogeneity among studies and the parameters of included studies could be pooled. There was no significant heterogeneity either in sputum induction studies (Cochran-Q of DOR = 5.55; *p* = 0.235) or in bronchoscopy studies (Cochran-Q of DOR = 2.71; *p* = 0.608). Therefore the SEN, SPE, PLR, NLR and DOR outcomes were pooled. In terms of mycobacterial culture, the SEN, SPE, PLR, NLR and DOR of sputum induction were 0.72(95%CI 0.66–0.77), 1.00(95%CI 0.99–1.00), 57.57(95%CI 16.89–196.30), 0.26(95%CI 0.17–0.42) and 280.08(95%CI 58.93–1331.3) respectively, whereas the SEN, SPE, PLR, NLR and DOR of bronchoscopy were 0.70(95%CI 0.64–0.75), 1.00(95%CI 0.99–1.00), 51.02(95%CI 14.93–174.32), 0.33(95%CI 0.26–0.42) and 166.43(95%CI 45.81–604.61) respectively(Table. 2, Fig. 3). Sputum induction had a similar AUC (0.9564, SE = 0.0749) with bronchoscopy (0.8618, SE = 0.1652) (*P* value for difference of AUC between sputum induction and bronchoscopy was 0.602 Fig. 4.a).

**Table 2:** Pooled results of sputum induction and bronchoscopy mycobacterial culture

	Pooled SEN	Pooled SPE	Pooled +LR	Pooled -LR	Pooled DOR
	(95%CI)	(95%CI)	(95%CI)	(95%CI)	(95%CI)
sputum induction	0.72	1.00	57.57	0.26	280.08
	(0.66–0.77)	(0.99–1.00)	(16.89–196.30)	(0.17–0.42)	(58.93–1331.3)
Broncho-scopy	0.70	1.00	51.02	0.33	166.43
	(0.64–0.75)	(0.99–1.00)	(14.93–174.32)	(0.26–0.42)	(45.81–604.61)

*SEN* sensitivities; *SPE* specificities; *+LR* positive likelihood ratio; *−LR* negative likelihood ratio; *DOR* diagnostic odds ratio

### Quantitative synthesis of acid-fast bacilli smear

Only three articles had enough data on acid-fast bacilli smear [5–7]. The Spearman correlation coefficient and *P* value of sputum induction (Spearman correlation coefficient: 0.500, *p*-value = 0.667) were same to bronchoscopy, both of which indicated that there was no significant threshold effect. There was no significant heterogeneity either in the sputum induction studies (Cochran-Q of DOR = 3.72; *p* = 0.156) or the bronchoscopy studies (Cochran-Q of DOR =5.84; *p* = 0.054). The SEN, SPE, PLR, NLR and DOR outcomes were pooled. The SEN, SPE, PLR, NLR and DOR of sputum induction were 0.35(95%CI, 0.29–0.42), 0.99(95%CI, 0.96–1.00), 12.72(95%CI, 2.14–75.76), 0.70(95%CI, 0.57–0.85) and 18.78(95%CI, 2.79–126.52) respectively, whereas the SEN, SPE, PLR, NLR and DOR of bronchoscopy were 0.38(95%CI, 0.32–0.45), 0.99(95%CI, 0.96–1.00), 11.92(95%CI, 1.27–112.26), 0.67(95%CI, 0.48–0.95) and 18.22(95%CI, 1.55–214.55) respectively(Additional file 1: Appendix S2). There was no significant difference on AUC between sputum induction (0.6016) and bronchoscopy (0.8163; *p* value for difference of AUC between them was 0.792 Fig. 4.b).

### Mycobacterial culture versus acid-fast bacilli smear of sputum induction in the diagnosis of pulmonary tuberculosis

In the three articles mentioned above [5–7], the mycobacterial culture was compared with acid-fast bacilli smear of sputum induction in the diagnosis of pulmonary tuberculosis. The SEN and SPE of mycobacterial culture were 0.66(95%CI, 0.60–0.72) and 0.99(95%CI, 0.98–1.00) respectively, whereas the SEN and SPE of acid-fast bacilli smear were 0.35(95%CI, 0.29–0.42) and 0.99(95%CI, 0.96–1.00) respectively. The SEN of mycobacterial culture was higher than acid-fast bacilli smear (*p* < 0.001).

### Sensitivity analysis

The combined AUC results were not materially altered after we sequentially excluded each study (Additional file 1: Appendix S3), suggesting that the results were not excessively dependent on a certain study.

### Publication bias

The Deeks’ funnel plot asymmetry test indicated no statistical evidence of publication bias in the sputum induction studies (*P* = 0.55). (Additional file 1: Appendix S4).

## Discussion

Diagnosis of tuberculosis is still a challenge for those sputum smear negative pulmonary tuberculosis. The purpose of this meta-analysis was to compare the diagnosis value of sputum induction and bronchoscopy in the diagnosis of sputum smear-negative pulmonary tuberculosis. We found that sputum induction had similar diagnostic performance with bronchoscopy in the diagnosis of sputum smear-negative pulmonary tuberculosis, both in terms of acid-fast bacilli smear and mycobacterial culture.

As is well known, bronchoscopy is an important examination for obtaining high quality respiratory specimens in pulmonary infectious disease and also recommended for the diagnosis of pulmonary tuberculosis [10, 15, 16]. Nevertheless, this technique is invasive, poorly tolerated and costly compared with sputum induction. Additionally, bronchoscopy is not easily available in resource-limited areas and it is not suitable in settings when a large quantity of people need to be evaluated.

Sputum induction is an uncomplicated, safe, cheap, and effective method for the diagnosis of pulmonary tuberculosis, which make it particularly suitable for being used in resource-limited settings [4, 17, 18]. Several studies have compared detection rates of sputum induction with bronchoscopy in tuberculosis cases. The results are variable and the sample size is relatively small [5, 9, 19]. Our meta-analysis showed that sputum induction had a similar overall diagnostic accuracy with bronchoscopy in sputum smear-negative pulmonary tuberculosis. Meanwhile, a prospective multicenter study showed that repeated induced sputum specimens could improve the diagnostic yield of pulmonary tuberculosis and it was not desirable to exclude the diagnosis of tuberculosis through a single specimen [19], which was in agreement with the recommendations of treatment of tuberculosis guidelines published by WHO [20]. Considering all of these, we suggest that for patients with suspected pulmonary tuberculosis who are smear-negative for acid-fast bacilli, sputum induction rather than bronchoscopy should be recommended as the initial method, which is in accordance with the guideline [10].

We compared the diagnostic yield of acid-fast bacilli smear with mycobacterial culture of sputum induction in pulmonary tuberculosis, and found that the SEN in mycobacterial culture was higher than in acid-fast bacilli smear, which is consistent with the previous study by Monkongdee et al. [21]. Our results are also in line with the guidelines for pulmonary tuberculosis diagnosis published by WHO [22]. So patients with suspected pulmonary tuberculosis who are acid-fast bacilli smear negative should undergo mycobacterial culture to increase the diagnostic yield of tuberculosis.

The limitations in our meta-analysis are as follows. First, the number of included studies was not large. This is because we only accepted studies that used sputum induction and bronchoscopy for detection of tuberculosis within the same population, which is also an advantage of this study because of the small heterogeneity. Second, since patients came from different places (some from primary health units and some from tertiary health care unit), the pretest probabilities of diagnosis of tuberculosis were different. Patients from tertiary health care unit had a higher pretest probability than those from primary health units, which could lead to potential heterogeneity. Third, the culture techniques in the different studies were different, but they were same for the culture of the two specimens (sputum induction and bronchoscopy specimen) in each study. So it didn’t significantly affect the heterogeneity of this study. Fourth, we only compared the diagnostic value of acid-fast bacilli smear or mycobacterial culture of sputum induction or bronchoscopy in tuberculosis, but were unable to compare the acid-fast bacilli smear joint mycobacterial culture of sputum induction or bronchoscopy, as there were no sufficient data of this topic for statistical analysis.

## Conclusions

Sputum induction has similar sensitivity, specificity and overall accuracy compared to bronchoscopy-obtained specimens in diagnosing for sputum smear-negative pulmonary tuberculosis. Meanwhile, mycobacterial culture has a higher sensitivity than acid-fast bacilli smear in diagnosing for sputum smear negative tuberculosis.

## Supplementary information

**Additional file 1: Appendix S1.** Full search terms used in the literature search of Embase. **Appendix S2.** Pooled results of sputum induction and bronchoscopy acid-fast bacilli smear. **Appendix S3.** Sensitivity analysis. **Appendix S4.** Publication bias.

## Data Availability

The dataset used and/or analyzed during the current study will be available from the corresponding author on a reasonable request after the final result is published in a journal.

## Abbreviations

SEN: Sensitivities
SPE: Specificities
PLR: Positive likelihood ratio
NLR: Negative likelihood ratio
DOR: Diagnostic odds ratio
CI: Confidence interval
AUCs: Areas under the curves
WHO: World Health Organization
PRISMA: The Preferred Reporting Items for Systematic Reviews and Meta-Analyses
TB: Tuberculosis
TP: True positive
FP: False positive
TN: True negative
FN: False negative
QUADAS-2: The Quality Assessment of Diagnostic Accuracy Studies
SE: Standard Error
