# Supplementary material for: Comparison of sputum induction and bronchoscopy in diagnosis of sputum smear-negative pulmonary tuberculosis: a systemic review and meta-analysis
Source: BMC Pulm Med. 2020 May 25;20:146. doi: 10.1186/s12890-020-01192-w (PMC7249394; doi:10.1186/s12890-020-01192-w)
Supplement: Supplementary file 1 — Additional file 1: Appendix S1. Full search terms used in the literature search of Embase. Appendix S2. Pooled results of sputum induction and bronchoscopy acid-fast bacilli smear. Appendix S3. Sensitivity analysis. Appendix S4. Publication bias. [file 12890_2020_1192_MOESM1_ESM.docx]

**SUPPLEMENTARY INFORMATION**

**Comparison of sputum induction and bronchoscopy in diagnosis of sputum smear-negative pulmonary tuberculosis:**

**a systemic review and meta-analysis**

Wen Luo^1^, Yihua Lin^1^, Zhibin Li^2^, Wanyu Wang^1^, Yonghong Shi^1^.

^1. Department of Pulmonary and Critical Care Medicine, the First Affiliated Hospital of Xiamen University, Xiamen, China.^

^2. Epidemiology Research Unit, the First Affiliated Hospital of Xiamen University, Xiamen, China.^

**^Grant Support:^** ^Youth Scientific Research Project (2016-2-64) of Fujian Provincial Health Commission and Xiamen Municipal Health Commission in China.^

**^Conflicts of Interests:^** ^None^

Appendix S1- Full search terms used in the literature search of Embase.

| ***Search ID*** | ***Search Terms*** |
| --- | --- |
| S10 | S3 AND S6 AND S 9 |
| S9 | S7 OR S8 |
| S8 | 'induced sputum'/exp OR 'induced sputum' OR (induced AND ('sputum'/exp OR sputum)) OR 'sputum induction'/exp OR 'sputum induction' OR  (('sputum'/exp OR sputum) AND ('induction'/exp OR induction)) OR 'induced sputa' OR (induced AND sputa) |
| S7 | 'sputum'/exp |
| S6 | S4 OR S5 |
| S5 | tuberculos* OR (pulmonary AND consumption*) OR 'pulmonary phthisis' OR (pulmonary AND ('phthisis'/exp OR phthisis)) |
| S4 | 'tuberculosis'/exp |
| S3 | S1 OR S2 |
| S2 | bronchoalveolar OR bronchoscop* |
| S1 | 'bronchoscopy'/exp |

| Appendix S2- Pooled results of sputum induction and bronchoscopy acid-fast bacilli smear. | | | | | |
| --- | --- | --- | --- | --- | --- |
|  | Pooled SEN | Pooled SPE | Pooled +LR | Pooled -LR | Pooled DOR |
|  | (95%CI) | (95%CI) | (95%CI) | (95%CI) | (95%CI) |
| sputum induction | 0.35 | 0.99 | 12.72 | 0.70 | 18.78 |
|  | （0.29-0.42） | （0.96-1.00） | （2.14-75.76） | （0.57-0.85） | （2.79-126.52） |
| broncho-scopy | 0.38 | 0.99 | 11.92 | 0.67 | 18.22 |
|  | （0.32-0.45） | （0.96-1.00） | （1.27-112.26） | （0.48-0.95） | （1.55-214.55） |

Appendix S3- Sensitivity analysis.

| First Author | sputum induction | |  | bronchoscopy | | *P* |
| --- | --- | --- | --- | --- | --- | --- |
|  | AUC | SE |  | AUC | SE |  |
| Anderson^(1)^ | 0.9572 | 0.0949 |  | 0.8631 | 0.1640 | 0.619 |
| Conde^(2)^ | 0.9372 | 0.1614 |  | 0.8738 | 0.1554 | 0.777 |
| McWilliams^(3)^ | 0.8831 | 0.1467 |  | 0.7774 | 0.2638 | 0.726 |
| Saglam^(4)^ | 0.9906 | 0.0167 |  | 0.9432 | 0.1146 | 0.682 |
| Prakash^(5)^ | 0.9570 | 0.1199 |  | 0.8108 | 0.2618 | 0.612 |

Appendix S4- Publication bias.


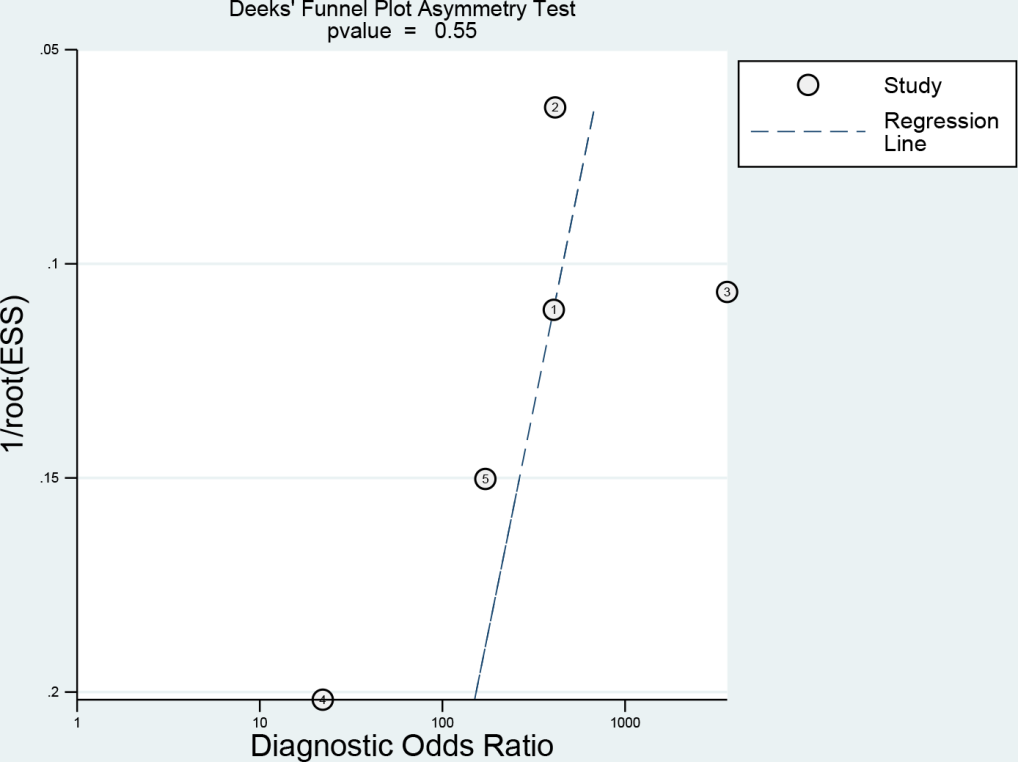


1. Anderson C, Inhaber N, Menzies D. Comparison of sputum induction with fiber-optic bronchoscopy in the diagnosis of tuberculosis. American journal of respiratory and critical care medicine. 1995;152(5 Pt 1):1570-4.

2. Conde MB, Soares SL, Mello FC, Rezende VM, Almeida LL, Reingold AL, et al. Comparison of sputum induction with fiberoptic bronchoscopy in the diagnosis of tuberculosis: experience at an acquired immune deficiency syndrome reference center in Rio de Janeiro, Brazil. American journal of respiratory and critical care medicine. 2000;162(6):2238-40.

3. McWilliams T, Wells AU, Harrison AC, Lindstrom S, Cameron RJ, Foskin E. Induced sputum and bronchoscopy in the diagnosis of pulmonary tuberculosis. Thorax. 2002;57(12):1010-4.

4. Saglam L, Akgun M, Aktas E. Usefulness of induced sputum and fibreoptic bronchoscopy specimens in the diagnosis of pulmonary tuberculosis. The Journal of international medical research. 2005;33(2):260-5.

5. Prakash P, Agarwal P, Gupta A, Gupta E, Dasgupta A. Comparison of Induced Sputum and Bronchoalveolar Lavage Fluid Examination in the Diagnosis of Sputum Negative Pulmonary Tuberculosis. The Indian journal of chest diseases & allied sciences. 2016;58(3):173-5.
